# Severe Mitral Regurgitation Diagnosis Through Point-of-Care Ultrasound During Initial Physical Examination

**DOI:** 10.7759/cureus.30150

**Published:** 2022-10-10

**Authors:** Evan M Cameron, Larry Istrail

**Affiliations:** 1 Internal Medicine, Inova Fairfax Medical Campus, Falls Church, USA

**Keywords:** mitral regurgitation, pocus, point-of-care ultrasound (pocus), point-of-care ultrasound, ultrasound imaging, physical diagnosis, comprehensive physical exam, mitral valve disease

## Abstract

Mitral regurgitation is a common valvular disorder found in the general population with varying degrees of severity. The symptoms of this disorder correspond to the severity of regurgitation as well as its associated complications such as arrhythmias. Suspicion of mitral regurgitation is based on physical exam findings with diagnosis generally requiring confirmatory findings on transthoracic echocardiogram. However, asymptomatic patients with mitral regurgitation and limited sensitivity of cardiac auscultation to detect a murmur confound the diagnosis. In this case, a patient presented with nonspecific symptoms of shortness of breath and abdominal pain in which a bedside point-of-care ultrasound (POCUS) in initial examination demonstrated severe mitral regurgitation and pulmonary edema. These findings expedited an intervention on the regurgitation, which highlights the importance of incorporation and early use of POCUS during physical examination.

## Introduction

Mitral regurgitation is prevalent in the general population and is characterized by the spectrum of pathophysiology and underlying severity. A trivial amount of regurgitation can be found in up to 66% of otherwise healthy individuals in what is known as physiologic mitral regurgitation [1]. Mild regurgitation is found in roughly 19% of the population [2] and the more progressive stages, moderate and severe, are found in 1.9% and 0.2%, respectively [3]. Symptoms correlate with the severity of regurgitation and are related to potential complications such as arrhythmias. It is important to note that symptoms can commonly present late in the disease process causing mild to moderate regurgitation to go undetected as patients may be asymptomatic.

A systolic murmur, more specifically an apical holosystolic murmur or late systolic murmur, should prompt suspicion of mitral regurgitation. The diagnosis is subsequently confirmed with a transthoracic echocardiogram. Other modalities such as a transesophageal echocardiogram or cardiovascular magnetic resonance imaging can be used if transthoracic echocardiogram imaging is suboptimal. The limiting factor in making the diagnosis lies in the physical exam as cardiac auscultation provides limited sensitivity for the detection of valvular disease [4-8]. Some studies suggest that the lack of sensitivity is due to auscultatory skills [5-7], whereas another study suggested that valve regurgitation detected by Doppler echocardiography frequently did not have a corresponding murmur on auscultation [8].

Point-of-care ultrasound (POCUS) during the physical examination can be used as a diagnostic modality to increase the diagnostic yield in mitral regurgitation. It has been demonstrated previously that handheld ultrasound is superior to standard physical examination in detecting substantial valvular disease [9]. Furthermore, POCUS significantly increases the diagnostic yield in cardiac abnormalities even in the less experienced clinician. One study demonstrated that handheld ultrasound used by medical students had a higher sensitivity and specificity in diagnosing cardiac abnormalities when compared to cardiologists using standard physical examination alone [10]. Due to the higher diagnostic yield in conditions with inconsistent physical examinations, POCUS should be incorporated into the physical examination.

## Case presentation

A 70-year-old female with a history of hypertension presented to the emergency room with complaints of abdominal pain, shortness of breath, and dizziness. Her symptoms had been present for four weeks prior to the presentation, which had prompted an abdominal MRI as an outpatient, which revealed a pancreatic cyst and a papillary mucinous neoplasm. On presentation, she was found to be hypoxic, requiring two liters of oxygen supplementation, and tachycardic to 108 beats per minute, with a blood pressure of 156/83 mmHg. On physical exam, she was found to have clear breath sounds, a nontender abdomen, and a systolic murmur on cardiac auscultation. Chest X-ray showed interstitial opacities, which were more pronounced in the lower lobe fields. Initial lab work was unremarkable.

POCUS performed during the initial physical exam revealed diffuse B-lines consistent with pulmonary edema (Video 1) and bilateral pleural effusion with compressive atelectasis. POCUS also demonstrated a preserved cardiac ejection fraction with severe mitral regurgitation and left atrial dilation (Videos 2, 3). These findings prompted treatment with intravenous diuretics for a new diagnosis of heart failure and a cardiology consultation for expedited evaluation and intervention on mitral regurgitation. A formal transthoracic echocardiogram confirmed severe mitral regurgitation due to posterior leaflet prolapse. Further workup included a transesophageal echocardiogram, which further elucidated the mitral regurgitation due to a flail P2 and P2-P3 prolapse. Right-sided heart catheterization demonstrated elevated right-sided filling pressures and an elevated wedge pressure. She was evaluated by cardiovascular surgery and ultimately underwent an open mitral valve repair.

**Video 1:** Pulmonary edema with B-lines seen on POCUS POCUS: point-of-care ultrasound.

**Video 2:** Parasternal long view displaying regurgitant flow

**Video 3:** Apical four-chamber view displaying mitral regurgitation

## Discussion

Mitral regurgitation is a common valvular disease that carries a range of symptoms that typically correlate with regurgitant severity [1-3]. Commonly, patients are asymptomatic until late in the disease process. Diagnosis of mitral regurgitation relies on a high degree of suspicion based on a physical exam, typically a murmur during systole, with confirmatory findings on formal echocardiography. The diagnosis of mitral regurgitation is at risk of being delayed or missed altogether due to the low sensitivity of cardiac auscultation to detect murmurs based on the skill of the physician or absence of murmur despite the disease state [4-8].

In this case, a patient presented with shortness of breath, which is a complaint that carries a broad differential, and was diagnosed with severe mitral regurgitation through POCUS on the initial physical exam in the emergency room. Though this patient may have ultimately been evaluated by a formal echocardiogram based on her symptoms and hypoxia, POCUS revealed urgent findings during the physical examination, which prompted an early intervention that may have been delayed when relying on cardiac auscultation alone.

The key to this case is recognizing that POCUS, when integrated into standard physical examination, can improve the detection rate of significant valvular abnormalities in the absence of an abnormal physical exam [9,10]. When not incorporating POCUS into the physical exam, there is a risk of delaying the diagnosis of mitral regurgitation until the patient becomes significantly symptomatic.

## Conclusions

Diagnosis of mitral regurgitation relies on a high degree of suspicion based on a systolic murmur on physical exam with subsequent echocardiogram for confirmation. Cardiac auscultation has a low sensitivity in detecting significant valvular abnormalities and allows the risk of delaying a diagnosis until a patient becomes significantly symptomatic. When incorporated with a physical exam, POCUS increases the rate of detection of significant mitral regurgitation independent of symptoms or abnormal physical exam.
